# Clinical usefulness of C-reactive protein to albumin ratio in predicting 30-day mortality in critically ill patients: A retrospective analysis

**DOI:** 10.1038/s41598-018-33361-7

**Published:** 2018-10-08

**Authors:** Tak Kyu Oh, In-Ae Song, Jae Ho Lee

**Affiliations:** 1 Department of Anesthesiology and Pain Medicine, Seoul National University Bundang Hospita, Seongnam, Korea; 2 Division of Pulmonary and Critical Care Medicine, Department of Internal Medicine, Seoul National University Bundang Hospital, Seongnam, Korea

## Abstract

This study aimed to examine the prognostic value of C-reactive protein (CRP)/albumin (ALB) ratio among patients who were admitted to the intensive care unit (ICU) in predicting 30-day mortality rate. This retrospective cohort study was conducted by examining the medical records of adult patients who were admitted to the ICU at Seoul National University Bundang Hospital between 1 January 2012 and 31 December 2016. Data from 6,972 individuals were included in the final analysis, and 547 of these individuals (7.1%) died within 30 days after their ICU admission. The multivariable Cox regression analysis revealed that an increase of 1 for the CRP/ALB ratio was associated with an 11% increase in the risk of 30-day mortality (hazard ratio: 1.11, 95% confidence interval: 1.09–1.14, *P* < 0.001). However, the area under curve of CRP/ALB ratio in receiver operating characteristic analysis was lower than that of Acute Physiologic Assessment and Chronic Health Evaluation (APACHE) II, Charlson comorbidity index, or serum albumin alone. Although an elevated CRP/ALB ratio on ICU admission was an independent risk factor for 30-day mortality rate, the predictive power of CRP/ALB ratio was lower than that of albumin alone, APACHE II, and Charlson comorbidity index.

## Introduction

C-reactive protein (CRP) and serum albumin (ALB) are useful markers that can predict morbidity and mortality among critically ill patients^1,2^. This is because CRP effectively reflects acute-phase inflammation^3^ while ALB may reflect malnutrition among critically ill patients^4,5^. The ratio of CRP to ALB (CRP/ALB) has also recently been used to predict the prognosis of patients with severe sepsis or septic shock^6,7^, with an increased CRP/ALB ratio at the intensive care unit (ICU) admission being independently associated with increased mortality rates. In addition, among patients who are receiving parenteral nutrition, the CRP/ALB ratio is closely related to morbidity and mortality^8^. Thus, the CRP/ALB ratio may be useful for evaluating critically ill patients and may address the weaknesses in other major scoring systems (e.g., the simplified acute physiology score 3 [SAPS 3], Acute Physiology and Chronic Health Evaluation II [APACHE] score, and Sepsis-related Organ Failure Assessment [SOFA]), which are not capable of directly evaluating malnutrition among critically ill patients^9,10^. Thus, as the CRP/ALB ratio effectively reflects both inflammation and malnutrition^11,12^, it may be a useful biochemical marker for predicting prognosis among critically ill patients. However, no studies have examined the general population of patients in the ICU to determine the relationship between the CRP/ALB ratio at their ICU admission and subsequent risk of mortality. We recently reported that the CRP/ALB ratio could be a useful prognostic factor for patients admitted surgical ICU after surgery^13^. However, the study did not assess the value of the CRP/ALB ratio compared with that of other prognostic factors such as APACHE II or Charlson comorbidity index.

Therefore, the present study aimed to examine the relationship between the CRP/ALB ratio and risk of 30-day mortality among all patients who were admitted to our centre’s ICU. In addition, the present study aimed to compare the CRP/ALB ratio with other prognostic factors (APACHE II, Charlson comorbidity index) in predicting 30-day mortality after ICU admission.

## Results

**Figure 1:**
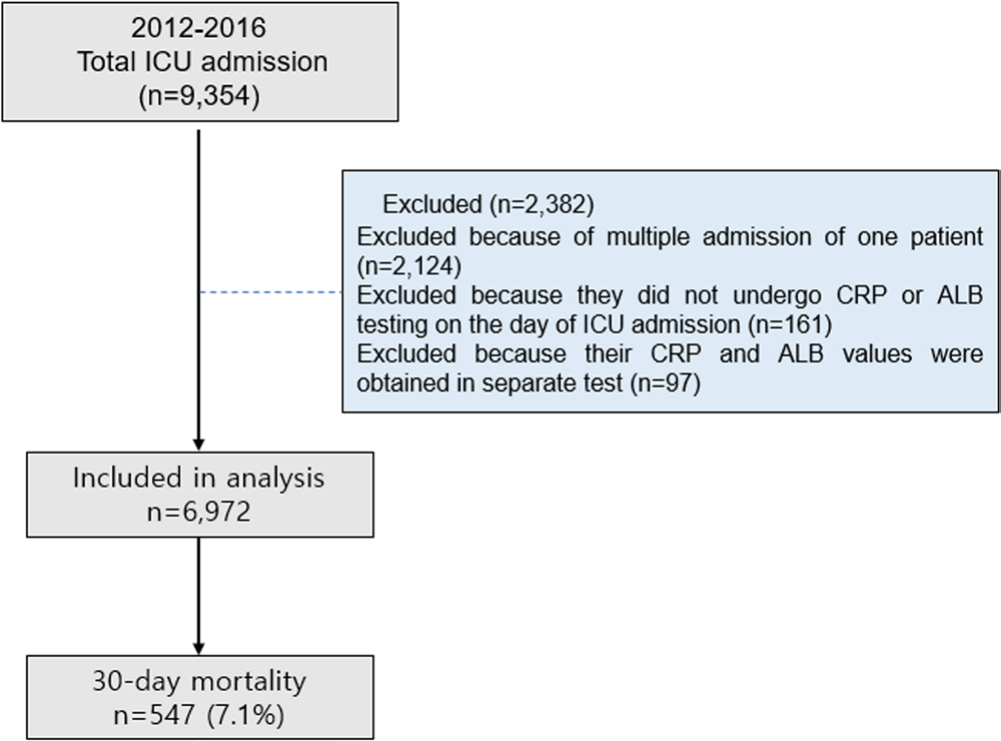
Flow chart of patient selection process ICU, intensive care unit; CRP, C-reactive protein; ALB, albumin.

Between 1 January 2012 and 31 December 2016, the Seoul National University Bundang Hospital (SNUBH) ICUs had 9,354 admissions, although 2,124 cases were excluded because of multiple admissions during the study period. Furthermore, 161 individuals were excluded because they did not undergo CRP or ALB testing on the day of their ICU admission. Another 97 individuals were excluded because their CRP and ALB values were obtained in separate tests. Thus, 6,972 individuals were included in the final analysis and 547 individuals (7.1%) died within 30 days after their ICU admission (Fig. 1 and Table 1).

**Table 1:** Baseline characteristics of patients who were admitted to ICU in 2012–2016.

Characteristic	Total patients (n = 6,972)	Mean	SD
Age, year		64.9	15.3
Sex: male	4,362 (62.6%)		
Body mass index, kg m^−2^		24.0	3.9
Postoperative admission	5,300 (68.8%)		
Department of admission: IM	1,520 (19.7%)		
Length of ICU stay, day		4.8	9.6
Length of hospital stay, day		35.4	68.7
APACHE II		21.7	8.3
Charlson Comorbidity Index		1.6	1.9
Comorbidity at ICU admission			
Hypertension	768 (10.0%)		
Diabetes mellitus	394 (5.7%)		
Ischemic heart disease	152 (2.2%)		
COPD	83 (1.2%)		
Cancer	1,478 (21.2%)		
Albumin (g/L)		30.8	5.3
C-Reactive Protein (mg/L)		73.4	66.3
C-Reactive Protein/Albumin ratio		2.6	2.6
30-day mortality after ICU admission	547 (7.1%)		

Presented as mean (SD) or Number (percentage).

CRP, C-reactive protein; ICU, intensive care units; IM, internal medicine; APACHE, acute physiology and chronic health evaluation; COPD, chronic obstructive pulmonary disease; SD, standard deviation.

### Risks of 30-day mortality after ICU admission

Tables 2 and 3 show the results of the univariable and multivariable Cox regression analyses for 30-day mortality after postoperative ICU admission. The multivariable Cox proportional hazard model revealed that an increase of 1 for the CRP/ALB ratio was associated with an 11% increase in the risk of 30-day mortality (hazard ratio [HR]: 1.11, 95% confidence interval [CI]: 1.09–1.14, *P* < 0.001; model 1). A 1 g/L increase of albumin was associated with a 13% decrease in the risk of 30-day mortality (HR: 0.87, 95% CI: 0.85–0.88, *P* < 0.001; model 2), while CRP was not associated significantly with 30-day mortality (*P* = 0.064; model 2). In addition, a 1 point increase in the APACHE II and Charlson comorbidity index was associated with a 7% (HR: 1.07, 95% CI: 1.06–1.08, *P* < 0.001) and 51% (HR: 1.51, 95% CI: 1.43–1.59) increase in the risk of 30-day mortality after ICU admission, respectively.

**Table 2:** Univariable Cox regression analysis in relation to 30-day mortality after ICU admission.

Variables	Univariable model	
	Hazard ratio (95% CI)	*P*-value
Sex: male (vs Female)	1.05 (0.89, 1.25)	0.581
Age, year	1.02 (1.01, 1.02)	<0.001
Body mass index, kg m^−2^	0.93 (0.91, 0.95)	<0.001
Postoperative ICU admission	0.59 (0.49, 0.70)	<0.001
Department of ICU admission: IM (Ref: Non-IM)	3.37 (2.85, 3.99)	<0.001
APACHE II	1.09 (1.08, 1.10)	<0.001
Charlson Comorbidy Index	1.34 (1.30, 1.38)	<0.001
Comorbidity at ICU admission		
Hypertension	1.46 (1.16, 1.85)	0.001
Diabetes mellitus	1.48 (1.09, 2.02)	0.012
Ischemic heart disease	1.37 (0.84, 2.26)	0.212
COPD	2.28 (1.34, 3.88)	0.002
Cancer	1.31 (1.08, 1.58)	0.006
C-Reactive Protein (mg/L)	1.01 (1.00, 1.01)	<0.001
Albumin (g/L)	0.87 (0.85, 0.88)	<0.001
C-Reactive Protein/Albumin ratio	1.11 (1.10, 1.13)	<0.001

ICU, intensive care units; IM, internal medicine; APACHE, acute physiology and chronic health evaluation; COPD, chronic obstructive pulmonary disease.

**Table 3:** Multivariable Cox regression analysis using stepwise backward elimination method in relation to 30-day mortality after ICU admission.

Variables	Multivariable model	
	Hazard ratio (95% CI)	*P*-value
Age, year	1.01 (1.00, 1.01)	0.034
Body mass index, kg m^−2^	0.95 (0.92, 0.97)	<0.001
Department of ICU admission: IM (Ref: Non-IM)	1.69 (1.39, 2.05)	<0.001
APACHE II	1.07 (1.06, 1.08)	<0.001
Charlson Comorbidy Index	1.51 (1.43, 1.59)	<0.001
Cancer	1.53 (1.26, 1.86)	<0.001
**C-Reactive Protein/Albumin ratio: model 1**	1.11 (1.09, 1.14)	<0.001
**C-Reactive Protein (mg/L): model 2**	1.00 (0.99, 1.00)	0.064
**Albumin (g/L): model 2**	0.87 (0.85, 0.88)	<0.001
Interaction: C-Reactive Protein * Albumin: model 2	1.00 (1.00, 1.00)	0.008

C-reactive protein/albumin ratio is included in another multivariable cox regression model (model 2) to avoid multicollinearity with C-reactive protein and albumin (VIF > 11.0).

ICU, intensive care units; IM, internal medicine; APACHE, acute physiology and chronic health evaluation; VIF, variance inflation factors.

### ROC analysis for 30-day mortality

**Figure 2:**
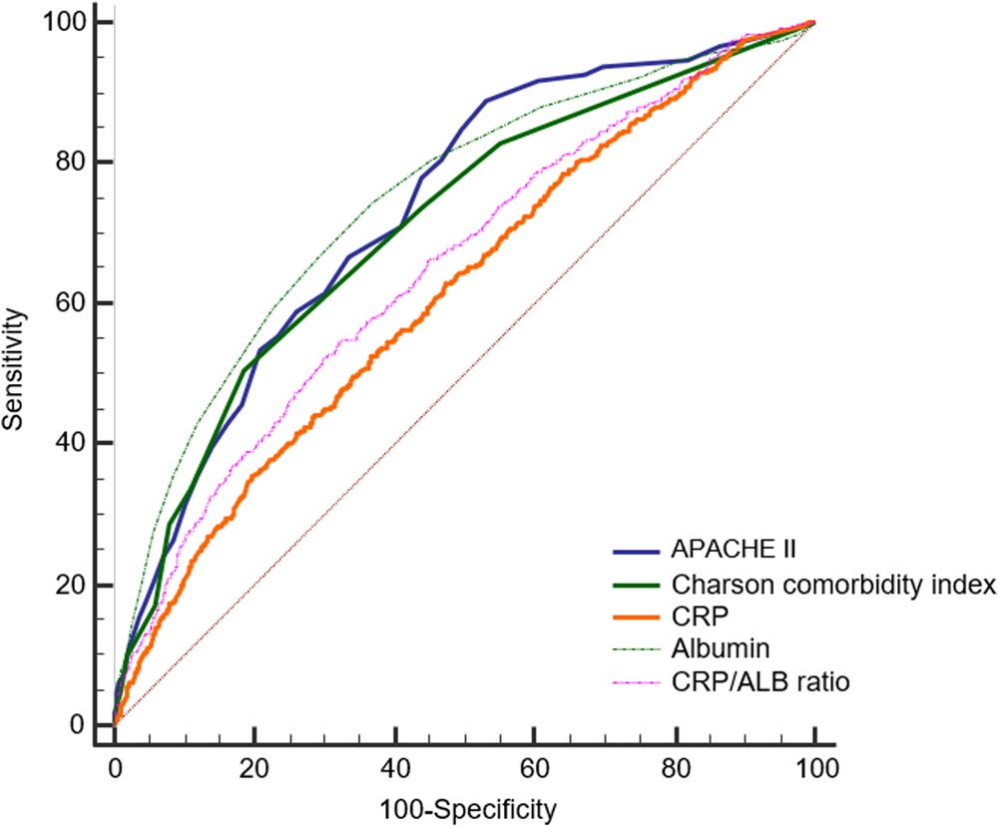
Receiver operating characteristic curve for 30-day mortality after intensive care unit admission. APACHE, acute physiology and chronic health evaluation; CRP, C-reactive protein; ALB, albumin.

Figure 2 shows the receiver operating characteristic (ROC) curve of each variable for 30-day mortality, and Table 4 shows the results of the area under curve (AUC) with 95% CI in ROC analysis for 30-day mortality. The AUC of the CRP/ALB ratio was 0.65 (95% CI: 0.64–0.66) which was significantly lower than the AUCs of APACHE II (0.73, 95% CI: 0.72–0.74) (Z = 5.22, *P* < 0.001) and the Charlson comorbidity index (0.71, 95% CI: 0.70–0.72) (Z = 3.53, *P* < 0.001). In addition, the AUC of albumin was 0.75 (95% CI: 0.73–0.76) which was significantly higher than that of CRP/ALB ratio (Z = 6.76, *P* < 0.001), while it did not significantly differ from the APACHE II (Z = 5.22, *P* = 0.116) or Charlson comorbidity index (Z = 2.41, *P* = 0.016).

**Table 4:** ROC analysis for 30-day mortality after ICU admission.

Variables	30-day mortality
	Area under curve (95% CI)
C-Reactive Protein (1)	0.61 (0.60, 0.63)
Albumin (2)	0.75 (0.73, 0.76)
C-Reactive Protein/Albumin ratio (3)	0.65 (0.64, 0.66)
APACHE II (4)	0.73 (0.72, 0.74)
Charlson comorbidiy index (5)	0.71 (0.70, 0.72)

Delong’s test, (1) vs (2): Z = 8.38, *P* < 0.001, (1) vs (3): Z = 12.79, *P* < 0.001, (1) vs (4): Z = 7.42, *P* < 0.001, (1) vs (5): Z = 5.68, *P* < 0.001, (2) vs (3): Z = 6.76, *P* < 0.001, (2) vs (4): Z = 5.22, *P* = 0.116, (2) vs (5): Z = 2.41, *P* = 0.016, (3) vs (4): Z = 5.22, *P* < 0.001, (3) vs (5): Z: 3.53, *P* < 0.001, (4) vs (5): Z = 1.57, *P* = 0.116.

ROC, Receiver operating characteristics; CI, Confidence Interval; Acute Physiologic Assessment and Chronic Health Evaluation II.

## Discussion

The present study revealed that an elevated CRP/ALB ratio at the ICU admission was independently associated with an increased risk of 30-day mortality. However, the predictive power of the CRP/ALB ratio was found to be significantly lower than that of the APACHE II or Charlson comorbidity index. Although the AUC of CRP/ALB ratio was significantly higher than that of CRP alone, AUC of albumin was higher than that of CRP/ALB ratio or CRP. As a result, this study reveals that using CRP/ALB ratio in predicting 30-day mortality after ICU admissions is not recommended instead of albumin alone, APACHE II, or Charlson comorbidity index. In other words, the clinical usefulness of the CRP/ALB ratio in predicting 30-day mortality is questionable.

To interpret results of this study, some points should be emphasized. First, we analysed the CRP/ALB ratio of a mixed ICU patient population while previous studies analysed a CRP/ALB ratio of more homogenous patient populations such those clearly diagnosed with sepsis^14^ or septic shock^6^. Considering that sepsis is accompanied by severe inflammation^15^, the impact of elevated CRP on 30-day mortality might be attenuated in this study. Secondly, although we included the comorbidity of cancer at ICU admission as a covariate, the proportion of cancer patients was 21.2% at ICU admission. Considering that elevated CRP is closely associated with a risk of cancer^16^, the impact of elevated CRP might also be attenuated in this study. Third, predictive value of serum albumin was so strong in critically ill patients^17^ that combining CRP with albumin (CRP/ALB ratio) was not beneficial in predicting 30-day mortality in critically ill patients.

Previous studies reported that the CRP/ALB ratio could be a useful prognostic factor in predicting mortality in patients with sepsis^14^, septic shock patients^6^, or critically ill patients requiring parenteral nutrition^8^. However, the previous studies did not evaluate the prognostic value of CRP/ALB ratio compared with that of albumin alone or other prognostic factors such as the APACHE II or Charlson comorbidity index^6,8,13,14^. By using the Delong test, we demonstrated that the ability of the CRP/ALB ratio for predicting mortality in critically ill patients is affected by a strong prognostic power of albumin at ICU admission. Furthermore, the prognostic value of a CRP/ALB ratio is not superior to that of traditional prognostic factors such as the APACHE II or Charlson comorbidity index.

The results of this study are similar to a previous study performed by Fairclough *et al*. who reported that a modified early warning system for acute medical admissions has better prognostic value for a patient’s outcome than the CRP/ALB ratio^18^. These researchers also suggested that pulse, respiratory rate, temperature, urine output, and systolic blood pressure were used for the modified early warning system. Considering that the APACHE II score includes heart rate and respiratory rate, the results of our study are similar to the results of Fairclough *et al*.^18^ In addition, we compared the difference in predictive power of 30-day mortality in critically ill patients between those assessed with the CRP/ALB ratio and Charlson comorbidity index, which are known to be useful indicators of chronic comorbidities of patients^19^.

The present study has several limitations. First, the retrospective single-centre design is associated with a risk of selection bias and/or limited generalizability of the results. Second, although patients were excluded if their CRP and ALB values were not measured during the same 24-h period, not all included patients underwent simultaneous testing of CRP and ALB. Lastly, since we analysed the mixed ICU patients in this study, applying the results of this study in specific population such as patients with cancer or sepsis is controversial. Nevertheless, the present study provides value, as it is the first study to analyse the relationship between the CRP/ALB ratio and 30-day mortality after ICU admission.

In conclusion, although an elevated CRP/ALB ratio at the ICU admission was an independent risk factor for 30-day mortality after ICU admission, the predictive power of CRP/ALB ratio is lower than albumin alone, APACHE II, and Charlson comorbidity index. Therefore, clinical usefulness of the CRP/ALB ratio in predicting 30-day mortality in critically ill patients is questionable.

## Methods

This retrospective cohort study was performed with the approval of the institutional review board of SNUBH (B-1806/474-105). The requirement for written informed consent was waived by the institutional review board; this manuscript adheres to the applicable STROBE guidelines.

Patients were included in the study if they were adults (≥19 years old) and were admitted to an ICU at SNUBH between 1 January 2012 and 31 December 2016. Only the last admission was considered for patients who were admitted to an ICU more than once during the study period. Patients were excluded if they did not undergo CRP and ALB testing on the same day as their ICU admission. This study is a sequential study of previous studies conducted by our institution^13^, that assessed patients who underwent postoperative ICU admission from 2007 to 2016, while this study analysed the patients who were admitted to all ICUs from 2012 to 2016.

The SNUBH has 1,360 beds and several ICUs with a total of 102 beds (Medical, Surgical, Neurologic, Emergency I and II). Each ICU has certified intensivists (anaesthesiologists, pulmonologists, neurologists, emergency physicians, and thoracic surgeons) that provide primary care during daytime office hours, while on-duty residents and fellows provide primary care at night and on weekends.

### Measurements and outcomes

Baseline data were collected regarding the patients’ demographic characteristics, history of underlying diseases, laboratory test results at the ICU admission, and exact dates of death. For example, the patients’ records were searched for diagnoses of hypertension, diabetes mellitus, coronary heart disease, chronic obstructive pulmonary disease, and cancer at their ICU admission. In addition, APACHE II scores and Charlson Comorbidity Indexes at the ICU admission were collected. All laboratory testing had been performed using venous or arterial sampling within 24 h after the ICU admission, and only the earliest test results were included if the same test was performed multiple times during the first 24 h. The Korean Ministry of the Interior and Safety approved the use of exact dates of death for all patients.

The primary outcome of interest was the relationship between the CRP/ALB ratio and 30-day mortality after ICU admission. In addition, the present study aimed to compare the CRP/ALB ratio with other prognostic factors (APACHE II, Charlson comorbidity index) in predicting 30-day mortality after ICU admission.

### Statistical analysis

Baseline characteristics of total patients were presented as numbers with percentages or means with standard deviations. First, we performed univariable Cox regression analysis to identify an individual relationship with 30-day mortality after ICU admission. Next, multivariable Cox regression analysis using a stepwise backward elimination method was performed to identify an independent relationship with 30-day mortality after ICU admission. In this multivariable Cox regression analysis, CRP, Albumin, and CRP/ALB ratio were included in another Cox regression model to avoid multicollinearity (variance inflation factors >11) within the model. All included variables fulfilled the Cox proportional hazard assumption based on a ‘log minus log plot’ with the CRP/ALB ratio.

Secondly, ROC analysis was performed to investigate predictability of CRP, albumin, CRP/ALB ratio, APACHE II, and Charlson comorbidity index. The AUC of the five variables were compared using Delong’s test^20^. All analyses were performed using IBM SPSS Version 23.0 (IBM Corp., Armonk, NY, USA), with *P*-values of <0.05 being considered statistically significant.

## Data Availability

The datasets used and/or analyzed during the current study are available from the corresponding author upon reasonable request.
